# Parliament and the Professions

**Published:** 1919-11-15

**Authors:** 


### PARLIAMENT AND THE PROFESSIONS.

### Qualification of Dentists.

It was stated that it is intended by the Government to introduce a Bill giving effect to the report of the Committee appointed to inquire into practice by unqualified dentists; but the issues involved are complex and numerous, and must be considered in relation to other necessary elements in the development of health services. No statement as to the precise lines of the Bill can be made.

### Pay of Nurses in Mesopotamia.

Col. Yate asked a question as to the effect of the rise in the exchange on nursing salaries; it appears that sisters receive 154 rupees instead of 231, and staff nurses 137 instead of 206, per month. He asked if the nursing staff could be paid at the fixed rate of 1s. 4d. to the rupee, as are non-commissioned officers and men. Mr. Forster replied that instructions were issued some time ago that the nursing service in Mesopotamia should receive, at the 1s. 4d. rupee, 10 per cent. of their pay, with the whole of their board, washing, and field allowance, as from April 1, 1919. This amount is ample to cover their necessary expenditure in Mesopotamia.

